# Antimicrobial Effects of Tetraspanins: A New Turnabout in Treatment of Microorganisms

**DOI:** 10.21315/mjms2022.29.4.2

**Published:** 2022-08-29

**Authors:** Khairiyah Murad, Sharaniza Ab Rahim, Hassanain Al-Talib

**Affiliations:** 1 Institute for Medical and Molecular Biotechnology (IMMB), Universiti Teknologi MARA, Selangor, Malaysia; 2 Department of Biochemistry and Molecular Medicine, Faculty of Medicine, Universiti Teknologi MARA, Selangor, Malaysia; 3 Department of Medical Microbiology and Parasitology, Faculty of Medicine, Universiti Teknologi MARA, Selangor, Malaysia

**Keywords:** tetraspanins, antimicrobial, bacterial adhesion, blocking peptide, antibodies

## Abstract

Tetraspanins, a transmembrane protein is a ‘molecular organiser’ with diverse functions that promote a network of signalling involved in the cellular and pathological processes. Tetraspanins-enriched microdomains (TEMs) are the crucial feature for the protein-protein interactions in the cell membrane and are vulnerable to pathogens exploitation. Targetting the tetraspanins has shown to be effective against microbial infections although more research is needed to be performed. This article will review previous evidence that has successfully demonstrated the potential mechanism to target tetraspanins as an antimicrobial agent.

## Introduction

Current studies revealed a rise in antimicrobial resistance within the human population. This has become a growing concern because it can inadvertently lead to other predicaments such as treatment failure and an increase in mortality. In 2014, O’Neill (1) made an astonishing prediction that approximately 10 million deaths globally will be recorded by the year 2050 due to antimicrobial resistance. The major causative factors for this phenomenon have been linked to the prolonged antimicrobial drugs and misuse of drugs, particularly antibiotics. The Ministry of Health (2) in Malaysia had reported that most bacteria develop resistance towards common antibiotics such as ciprofloxacin, cefotaxime and ampicillin ranging from 10% to 50% among different bacterial species. Therefore, it is imperial to discover and plan new strategies as effective treatments to combat microbial resistance. An alternative method has been proposed to target the protein or system of the host organism that can inhibit microbe adhesion and invasion without any development of microbial resistance.

This review article will emphasise the effects and relevance of targetitng tetraspanins as potential targets for the design and development of a targeted treatment in reducing microbial pathologies especially bacterial adhesion and viral entry to the host cell. The review consists of a short and brief overview of tetraspanins, locations, their structure and modifications for a better understanding of routes in targeting tetraspanins.

## Tetraspanins

Tetraspanins are a family of transmembrane proteins composed of 200–350 amino acids spanning the membrane four times and are embedded up to 5 nm above the membrane of multicellular eukaryotes (3). There are 33 identified mammalian members of tetraspanins that can be found typically in all cell types which could be used to indirectly promote antimicrobial properties together with other therapeutic agents (4). Different tetraspanin family members showed a different level of expression depending on the location of the distribution. It is either restricted to only a specific cell or tissue or broadly expressed across the cells or tissues explaining the reasons some tetraspanins are well-studied while some are understudied (5). The tetraspanin cell surface glycoproteins of CD9 (TSPAN29), CD63 (TSPAN30), CD81 (TSPAN28) and CD151 (TSPAN24) are the most known members of the tetraspanins family. Tetraspanins are associated and interact laterally with different types of proteins, lipids and other tetraspanins members for numerous fundamental cellular and pathological functions.

Tetraspanins can interact with different types of proteins such as adhesion molecules, immunoglobulin, signaling receptors, intracellular signaling molecules and immune signaling molecules (3, 6). Tetraspanins also can interact with lipids namely membrane gangliosides and cholesterol (3, 7). The web of tetraspanins allows it to form larger downstream complexes. These molecular networks promote cell adhesion, motility, fusion, membrane trafficking, signalling and as the key access to infectious diseases (3, 5, 8). The major disadvantage is that although tetraspanins can be found in all cell or tissue types, the specific functions of each tetraspanin through the interactions with other tetraspanins in tetraspanins-enriched microdomains (TEMs) remain ambiguous. This further clarifies their functional redundancy.

### Location of Tetraspanins

Tetraspanins are generally found on all human cells located on the cell surface or in the intracellular compartments such as in the lysosome and endosome (6). Studies reported that CD9 and CD81 are predominantly located at the cell surface while CD63 and CD151 are located intracellularly (9–10). Tetraspanins that are located on the cell surfaces tend to undergo less rate of recycling compared to tetraspanins that are located intracellularly that have a higher rate of internalisation (10). A summary of genetic analysis of tetraspanins molecule including chromosome localisation and molecular weight was obtained in a review article published by Rubinstein and Boucheix in 2009 (6).

### Structure and Modifications of Tetraspanins

**Figure 1:**
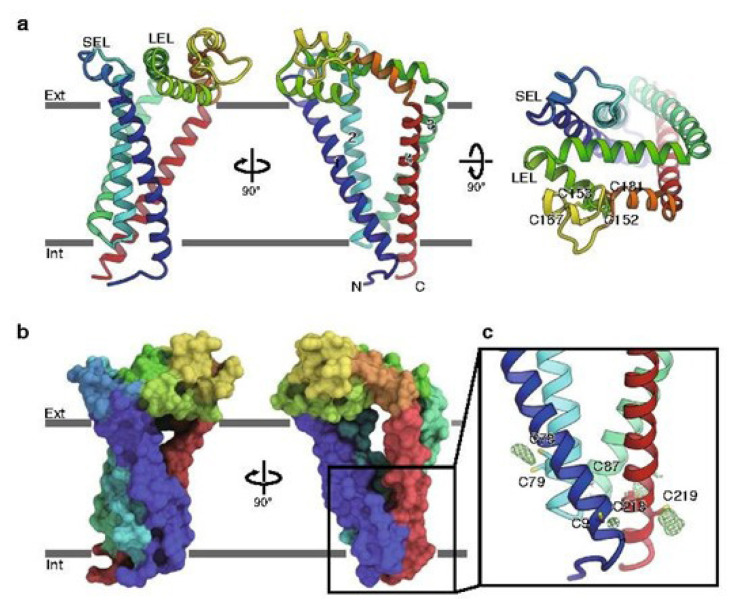
The CD9 (representative) (a) overall structure of the tetraspanins, (b) surface expression of the tetraspanins and (c) location of palmitoylation at the cysteine residues (11)

The basic structural components of tetraspanins consist of four hydrophobic transmembrane domains with cytoplasmic and extracellular loops alongside intracellular N- and C-termini (3, 6). The extracellular domains are made of small extracellular loops called EC1/SEL containing 13–31 amino acids and large extracellular loops called EC2/LEL containing 69–132 amino acids (5) (Figure 1). The EC2 is known to be the ‘signature’ structural component of tetraspanins where most of the protein-protein interactions occur and is also a common target for anti-tetraspanins monoclonal antibodies (3). The EC2 consists of two sub-domains of one constant region with 3-alpha helices and one hypervariable region (3, 6). The EC2 also comprised of conserved Cys-Cys-Gly amino acid motif (CCG motif) and another two conserved cysteine residues within the loop (3, 8).

Tetraspanins can homo- or hetero-dimerise with another tetraspanin or to other proteins and lipids to form TEM in the cell membrane (2). Signal transductions and all transmembrane signallings are expressed in the TEM region, hence explaining the ‘molecular organiser’ or the ‘tetraspanins web’ term (3, 9). Tetraspanins undergo modifications at the cysteine residue within the transmembrane domains, mainly through palmitoylation as shown in Figure 1 while some undergo ubiquitination or glycosylation (8). These modifications facilitate the tetraspanins membrane organisation, stabilisation, modulation and downregulation of the tetraspanins-partner interactions (3, 6, 8).

### Antimicrobial Properties of Tetraspanins

It has been reported that tetraspanins are involved in the pathogenesis of viral and bacterial infections, fungi, protozoa infections and as the initial microbial binding partners (5). They are known to be the ‘gateway’ for infections through their TEM-dependent complexes at the EC2 region (5, 11). Tetraspanins are utilised directly or indirectly by the pathogens to manipulate the normal cellular processes for pathogens binding, intracellular trafficking, replication, and infection development within the human cells (7). Tetraspanins are well documented to be associated with the hepatitis C virus and human immunodeficiency virus (HIV) for viral entry into the host cells (5). CD81 has been shown to act as a co-receptor or a ligand for the hepatitis C virus by incorporating into the envelope protein (E2) of the hepatitis C virus for viral entry and in the post-binding stage (5, 11). Previous studies have reported that tetraspanin-mediated adhesion is the initial step for the bacterial in invading the host cells and breaching the host cells barrier. Uroplakin 1a (TSPAN21) and Uroplakin 1b (TSPAN20) have been found to attach to the type 1 fimbriae, *Fim*H adhesin of uropathogenic *Escherichia coli* that invaded the bladder epithelial cells leading to urinary tract infections in humans (5, 7). The involvement of tetraspanins in viral infections has been reviewed since 2005 by Martin and the team (12). In addition, Van Spriel and Figdor (5) and Monk and Partridge (11) have conducted extensive research to determine the role of tetraspanins in diseases caused by various bacteria, viruses, protozoa and fungi. Targetting the tetraspanin receptors that are essential for microbial adherence, entry, trafficking and fusion inhibitory mechanism will interfere with tetraspanins binding site or functional complexes with the partner proteins of the microbes (13).

### Tetraspanins Monoclonal Antibodies

A previous study by Nishiuchi et al. (14) presented a specific anti-tetraspanin antibody called anti-CD151 that reduced the ability of tetraspanin-mediated adherence as it modulates the tetraspanins normal function. Anti-CD151 helps in dissociating CD151 from integrin α3β1 through the ‘lock and key’ or active conformation theory (14). Anti-CD151 can bind at or near the integrin-binding site and reducing the affinity for CD151, hence preventing CD151-α3β1 interaction and induced structural changes for any bacterial adherence (14). Antibodies against CD63 and CD9 showed a reduction in *Neisseria meningitidis* adherence to the epithelial cell line, however, it was not significant in anti-CD151 (10). The combination of anti-tetraspanin monoclonal antibodies (anti-CD9, anti-CD63 and anti-CD151) has been shown to significantly reduced the bacterial cell adherence of *Neisseria lactamica, Escherichia coli* and *Streptococcus pneumoniae* (10). Besides, a single treatment of anti-CD63 and anti-CD81 antibodies has been shown to effectively reduced the binding of *Salmonella typhimurium* with human monocyte-derived macrophages cells (15). This explains the importance of tetraspanin-specific antibodies in blocking the lateral associations with the partner proteins and disorganising the adhesion platforms required for bacterial adherence by targetting the pathogen indirectly. It was reported that anti-CD63 antibodies showed significant effects against *Salmonella typhimurium* at low concentrations since there were no significant effects observed using a high concentration at 100 μg/mL (15). In an earlier report by Green and co-workers (10), a combination of all tetraspanins monoclonal antibodies added no extra adherence inhibitory effect on *Neisseria meningitidis* to the target cells. Overall, these observations provide a strong indication that the anti-tetraspanins antibodies could be effective at a small concentration range. Furthermore, the team also addressed the use of antigen-binding fragments (Fab fragments) as an adherence inhibitor and successfully demonstrated a reduction in bacterial adherence. However, their findings were not in agreement with the results from a study by Hassuna et al. (15) whereby the researcher found no significant adherence inhibition effect by Fab fragments. The researcher also indicated that bacterial adherence inhibition required antibody cross-linking with tetraspanins and adhesion molecules.

### Recombinant Soluble Extracellular Loops

Small extracellular loop peptide (EC1/SEL) and/or large extracellular loop peptide (EC2/LEL) can inhibit the E2 protein of hepatitis C virus binding with tetraspanins (13). This inhibitor molecule can disturb the binding site of the tetraspanins to be associated with the pathogens due to the affinity changes. Interestingly, Green and his colleagues (10) stated that the recombinant glutathione S-transferase-large extracellular loops (GST-EC2) treatments of GST-EC2 CD63 and GST-EC2 CD151 were more effective even in a single treatment to lower down the bacterial adherence of *Neisseria lactamica, Escherichia coli*, *Streptococcus pneumoniae* and *Staphylococcus aureus* compared to the single-specific-anti-tetraspanin antibodies. A weak inhibitory effect was also reported using a recombinant protein LEL-GST EC2 of CD63, CD151, CD81 and CD9 against human papillomavirus (HPV) (16) on the immortalised human epithelial cells (HeLa) and immortalised human keratinocytes (HaCaT), while no significant reduction was observed in human cytomegalovirus (HCMV) infections using human umbilical vein endothelial cells (EA.hy926) (16). Short synthetic peptides derived from the EC2 of tetraspanin CD9 were used by Ventress et al. (17) at a low concentration (as low as nM) to sufficiently inhibit *Staphylococcus aureus* adherence to human keratinocytes. However, no notable inhibitory effect was observed at concentrations below 0.5 nM (17). These data suggested the mechanism of action that disrupts the TEM complex, reduces the TEM functionality and hence interferes with the tetraspanins-partner interactions within the membrane.

### Cytoplasmic C-Terminal Tail Peptide of Tetraspanins

The cytoplasmic C-terminal tail of tetraspanins is an essential structure of tetraspanins that play an important role in bacterial-host adhesion activity. The cytoplasmic C-terminal end is important for the cytoplasmic protein-protein interactions, in which inhibiting this region or using a mimic agonist molecule will reduce the ability for endocytic viral entry into the host cells (16). A previous study demonstrated that C-terminal peptides of CD63, CD151 and CD81 reduced the infection of HPV using HeLa cells and HaCaT cells while C-terminal peptides of CD63 and CD151 reduced the HCMV infection in vivo through EA.hy926, immortalised human endothelial cell-large T antigen and telomerase (HEC-LTT) and human foreskin fibroblast (HFF) cells as the model of infection (16). The study also reported that C-terminal peptides transfected with an enhancer (protein delivery reagent PULSin) showed an elevated inhibition effect against HPV infection. This is due to the amplification of peptides delivery into the cytoplasmic region which then increased the effectiveness of the treatment (16). However, further research is needed to ascertain the inhibitory role of C-terminal peptides against microbial infections.

### RNA Interference as a Potential Tetraspanins Inhibitor

The function of tetraspanins is also inhibited by the downregulation of tetraspanins through RNA interference (RNAi) using small-interfering RNA (siRNA) knockdown. Knocking down the tetraspanins would be beneficial to block the functionality of tetraspanins to reduce the entry of pathogens to the host cells (18). Evidence showed that a siRNA of CD151 successfully knockdown the expression of CD151 up to 90% and this consequently reduced the cell adhesion activity of the tetraspanins on the cells (14). Knockdown of siRNA CD63 was also reported to significantly reduced the *Salmonella typhimurium* binding to human macrophage cells by 50% compared to the control cells (15). Similar findings were reported by Green et al. (10) and Fast et al. (16) in which the siRNA of CD9, CD63 and CD81 produced a reduction in *Neisseria meningitidis* adhesion to the epithelial cells and HPV infections on HeLa cell lines.

## Effect of Anti-Tetraspanin Peptides on Host Cells

Using anti-tetraspanins peptides revealed no negative effect on the host cells’ viability, cell migration, and no effect on the bacterial internalisation, growth and viability (10, 17). Nevertheless, some of the bacterial cells were not affected in any of the treatments and this could be explained by the tetraspanin-independent mechanism of bacterial adherence and viral endocytosis into the host cells (10, 15, 17). Surprisingly, the anti-tetraspanins antibodies on *Neisseria meningitidis* lacking the gene for adhesins of Opa and Pili exhibited little to no significant effect in reducing bacterial adherence (10). This condition could explain that the tetraspanins are the ‘facilitator’ for the rapid microbial infections development onto the host cells.

## Future Direction and Its Application

At the end of 2019, the world was hit by a pandemic of coronavirus disease known as COVID-19 that is symptomatic of severe pneumonia, difficulty in breathing and some cases, diminished olfactory or gustatory senses (19). The extracellular vesicle (EVs) are released from many eukaryotic cells to remove any unwanted compounds or waste products from the cells (20). The EVs promote the pathogenesis of different viral diseases including COVID-19 by transferring the viral particles from the infected cells to the healthy cells (20–21). Specific COVID-19 virus EVs are involved in the binding to the host cells and are found abundantly within the tetraspanins, mainly the TEM region (20). Urciuoli and Peruzzi (19) suggested inhibiting the EVs trafficking as an approach to inhibit the virus infection. Disrupting the TEM region might be a new alternative to inhibit the virus budding and spreading hence, hindering COVID-19 infection. A study in 2015 found that TEM incorporated with the COVID-19 virus receptors and priming proteases indicated that the viral entry is mostly happening in the TEMs to facilitate the COVID-19 entry to the host cells (22). Tetraspanin monoclonal antibodies (anti-CD9, anti-CD63 and anti-CD81) have been demonstrated to inhibit the COVID-19 entry stage. Incubation of the antibodies at 30 min before virus inoculation showed no effect suggesting that anti-tetraspanin antibodies inhibit virus entry into the host cells within the TEM, hence reducing the infection (22). This strategy should be adopted by the researcher as a prophylactic measure alongside vaccination as part of the preventive and control measure of COVID-19.

## Current Application in Malaysia

There was a lack of study on the molecular interactions of multidrug-resistant bacteria with tetraspanin. The association between tetraspanin and adherence inhibition properties against bacteria and viruses is not yet established in Southeast Asian countries including Malaysia. Similarly, whether the tetraspanin could minimise bacterial and viral infections via the application of recombinant tetraspanin or anti-tetraspanin antibodies without the development of bacterial resistance is not yet determined. Nevertheless, this has led us to be the first in Malaysia to carry out a study investigating the antimicrobial effect of tetraspanin CD9 against *Pseudomonas aeruginosa* since *Pseudomonas aeruginosa* infections are very challenging to eradicate due to antimicrobial resistance against most conventional antibiotics.

## Conclusion

These approaches may offer an alternative to the current antimicrobial treatments. This is because they are directed to the host cell processes by disrupting the platforms for the bacterial attachments and viral trafficking into the host cells than directly aiming the pathogens. More research should be performed by engaging a broad range of bacteria and viruses including multidrug-resistant-pathogens and more information should be gathered on other anti-tetraspanin molecules. Further studies need to look more closely at the effect of the disrupted TEM complex, disrupted in the palmitoylation of tetraspanins and the time-dependent effect of the anti-tetraspanins incorporating the live-cell imaging studies. The use of in vitro and in vivo model organisms such as 3D-tissue engineered skin cells would be beneficial to explore the effectiveness of the treatment to be used in the clinical setting.

**Highlights**

- Tetraspanins are known as a ‘molecular organiser’, ‘tetraspanin web’, and a ‘gateway’ to infection through the TEMs.
- Targeting the tetraspanins is a targeted treatment to reduce bacterial adhesion and viral entrance to the host cells.
- Tetraspanins may be targeted using monoclonal antibodies, recombinant soluble EC2/LEL, cytoplasmic C-terminal tail peptides and siRNA that block the tetraspanin-pathogen interactions. This action will diminish the bacterial adhesion and viral entrance to the host cells.
